# Asphaltite Ash as a Mineral Filler in Bitumen: Physical, Rheological and Microstructural Assessment

**DOI:** 10.1021/acsomega.6c09051

**Published:** 2026-10-08

**Authors:** Ozgur Ozcan, Ahmet Saygin Tatar, Sedat Ozcanan, Abdulgazi Gedik

**Affiliations:** † Department of Civil Engineering, 187477 Sirnak University, M. Emin Acar Campus, Sirnak 73000, Turkey; ‡ Department of Civil Engineering, Faculty of Engineering and Natural Sciences, 531771 Malatya Turgut Ozal University, Malatya 44900, Turkey; § Graduate of Transportation Engineering, Faculty of Civil Engineering, Istanbul Technical University, Istanbul 34469, Turkey

## Abstract

Mining wastes represent a significant environmental challenge due to their large volumes and limited reuse potential. This study evaluates combustion-derived asphaltite ash (AA) as a mineral filler in bituminous binders through physical, rheological, thermal, chemical, and microstructural characterization. AA was produced by laboratory combustion of asphaltite at 1000 °C and incorporated into a B 50/70 binder at contents of 0, 2, 4, 6, and 8 wt % relative to the initial mass of the base binder. Penetration, ductility, and elastic recovery decreased, whereas the softening point increased with AA incorporation. Rotational viscosity decreased at 135 °C but increased at 165 °C relative to the control at the same temperature. Among the tested contents, AA4 exhibited the highest unaged rutting parameter and failure temperature of 78.9 °C, compared with 77.3 °C for the neat binder. However, AA4 also showed the highest stiffness to *m*-value ratio at −12 °C, indicating a less favorable low-temperature response. SEM–EDX and FTIR observations support a predominantly physical filler action associated with particle dispersion and mineral-phase incorporation, without demonstrating chemical bonding or cross-linking. AA4 exhibited the highest measured thermal degradation temperatures, while the residual mass increased with AA content because of the inorganic nature of the filler. These findings classify AA as a mineral filler and identify 4% AA as a candidate content for further high-temperature evaluation, rather than as a general optimum.

## Graphical abstract

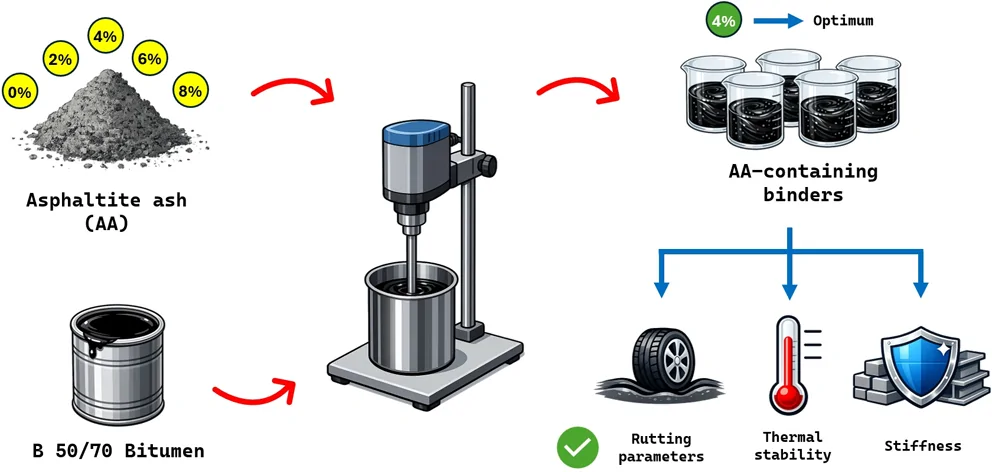

## 1 Introduction

Mining and energy-production activities generate considerable quantities of mineral residues whose long-term storage may create environmental and land-use concerns. The reuse of these materials in pavement applications may provide an alternative waste-management route while reducing the consumption of conventional mineral resources.^1–3^ At the same time, the temperature-dependent viscoelastic behavior of bitumen can adversely affect pavement performance under different climatic and loading conditions.^4^ Although conventional polymers are widely used to improve binder properties,^5,6^ their relatively high cost and environmental burden have encouraged the investigation of waste-derived and mineral-based alternatives.^7,8^

Among these alternatives, mineralogical and mining wastes have been investigated primarily as substitutes for conventional mineral fillers or fine aggregate fractions in asphalt materials. Steel slag, phosphate mining residues, coal fly ash, red mud, copper slag, and other mineral wastes have shown the potential to increase stiffness, stability, and resistance to permanent deformation.^9–11^ In binder–filler systems, these effects are generally associated with the physical presence of rigid mineral particles, increased particle–binder contact, absorption of lighter binder fractions, and restriction of molecular mobility. However, the resulting behavior depends strongly on mineral composition, particle size, surface morphology, and dosage. Excessive filler contents may increase viscosity and brittleness or promote particle agglomeration, thereby adversely affecting workability and low-temperature performance.^12,13^ Therefore, the effects of each waste-derived filler should be evaluated over an appropriate dosage range and under both high- and low-temperature conditions.

Asphaltite is a naturally occurring solid hydrocarbon formed through advanced stages of petroleum metamorphism and is characterized by its high asphaltene content and substantial inorganic fraction.^14,15^ Significant asphaltite deposits occur in southeastern Turkey, particularly around Şırnak, Siirt, and Mardin, with an estimated total reserve of approximately 77.5 million tons. Şırnak asphaltite is distinguished by its relatively high ash content, ranging from approximately 35% to 42.7%.^14,16^ This inorganic fraction consists mainly of calcite, dolomite, pyrite, and quartz, together with smaller amounts of clay minerals such as Illite and gypsum.^16^

Natural asphaltite and gilsonite have previously been investigated as bitumen modifiers because their organic and asphaltenic components can interact with the binder and alter its rheological behavior.^17–19^ The available studies generally indicate that these materials increase binder stiffness and improve resistance to permanent deformation at elevated temperatures, although excessive stiffening may reduce fatigue or low-temperature cracking resistance.^20–22^ Some studies have also reported that natural asphaltite may influence the aging behavior of bitumen through its organic constituents.^23^ However, these findings cannot be directly transferred to asphaltite ash. Unlike natural asphaltite, combustion-derived asphaltite ash (AA) has lost most of its combustible organic fraction and consists predominantly of inorganic mineral particles. Consequently, AA should not be assumed to act as a chemical modifier; its contribution is more appropriately evaluated in terms of physical mineral-filler effects.

Although the direct use of natural asphaltite in bituminous binders has been examined in previous studies, information regarding the binder-scale behavior of its combustion-derived ash remains limited. In particular, the dosage-dependent effects of AA on consistency, workability, high- and low-temperature rheological behavior, thermal response, and particle distribution have not been sufficiently established. It is also necessary to determine whether the observed changes originate from chemical modification or from the physical interaction between the binder and mineral particles. Addressing this distinction is important for defining the appropriate terminology, incorporation method, and potential practical use of AA.

Accordingly, the present study evaluates AA as a mineral filler in B 50/70 bituminous binder. AA was incorporated at contents of 2%, 4%, 6%, and 8% by mass of the base binder. Its effects were investigated using conventional physical tests and rotational viscosity measurements, together with Dynamic Shear Rheometer (DSR) and Bending Beam Rheometer (BBR) analyses. Scanning Electron Microscopy Coupled with Energy-Dispersive X-ray Spectroscopy (SEM–EDX) and Fourier Transform Infrared Spectroscopy (FTIR) analyses were used to examine particle distribution, elemental composition, and the nature of the interactions between AA and the binder, while Differential Scanning Calorimetry (DSC) and Thermogravimetric Analysis (TGA) were employed to characterize thermal behavior. The objectives were to quantify the dosage-dependent effects of AA, examine the trade-off between high-temperature stiffening, workability, and low-temperature response, and determine whether its predominant action is consistent with that of a physically interacting mineral filler. The scope of the study is limited to unaged binder-scale behavior and does not establish aging resistance, stability, or asphalt-mixture performance.

### 1.1 Statement of Novelty

Natural asphaltite has previously been investigated as a bitumen modifier because it retains hydrocarbon and heavy organic fractions that may contribute to physicochemical interactions with the binder. The material evaluated in the present study differs fundamentally from natural asphaltite because it was produced by combustion at 1000 °C, which removed most of the organic constituents and resulted in a predominantly inorganic, mineral-rich ash. Accordingly, AA is classified in this study as a mineral filler rather than as a chemical modifier. The novelty of the work lies in the systematic binder-level evaluation of combustion-derived asphaltite ash through physical, rheological, thermal, and microstructural characterization. The combined results clarify the dosage-dependent physical filler action of AA and distinguish its behavior from that of natural asphaltite used in its unburned state.

## 2 Materials and Methods

### 2.1 Materials

#### 2.1.1 Base Binder

B 50/70 penetration grade bitumen, supplied by the Batman refinery of the Turkish Oil Refineries Corporation (TÜPRAŞ), was used as the base binder. Prior to AA incorporation, the binder was characterized in accordance with EN 12591 ^24^ to verify compliance with specification limits. Physical properties including penetration, softening point, ductility, specific gravity, flash point and mass loss were determined to establish baseline performance characteristics. The determined values are given in Table 1, and as shown, all measured values fall within the specified standard limits.

**Table 1:** Physical Properties of the Neat Bitumen

Characteristic	Value	Criteria (EN 12591)
Ductility at 25 °C (cm)	150	≥100
Flash Point (°C)	279	≥230
Mass Loss (%)	0.18	≤0.50
Penetration at 25 °C (0.1 mm)	51.5	50–70
Penetration Index (PI)	–0.17	-
Softening Point (°C)	53.7	46–54
Specific Gravity at 25 °C	1.0185	1.01–1.06

#### 2.1.2 Asphaltite Ash (AA)

The asphaltite used in this study was obtained from asphaltite deposits located in the Şırnak–Avgamasya region of Turkey, as shown in Figure 1. The study area is situated within a well-known asphaltite-bearing zone characterized by the presence of multiple vein-type deposits distributed along the regional geological formations. These veins are observed in proximity to local settlements such as Milli, Nivekara, and Harbul, and are spatially associated with the drainage network, including the Hezil and Nerduş rivers. The Avgamasya deposit, highlighted as the primary sampling location, represents one of the significant asphaltite occurrences in the region.

**Figure 1:**
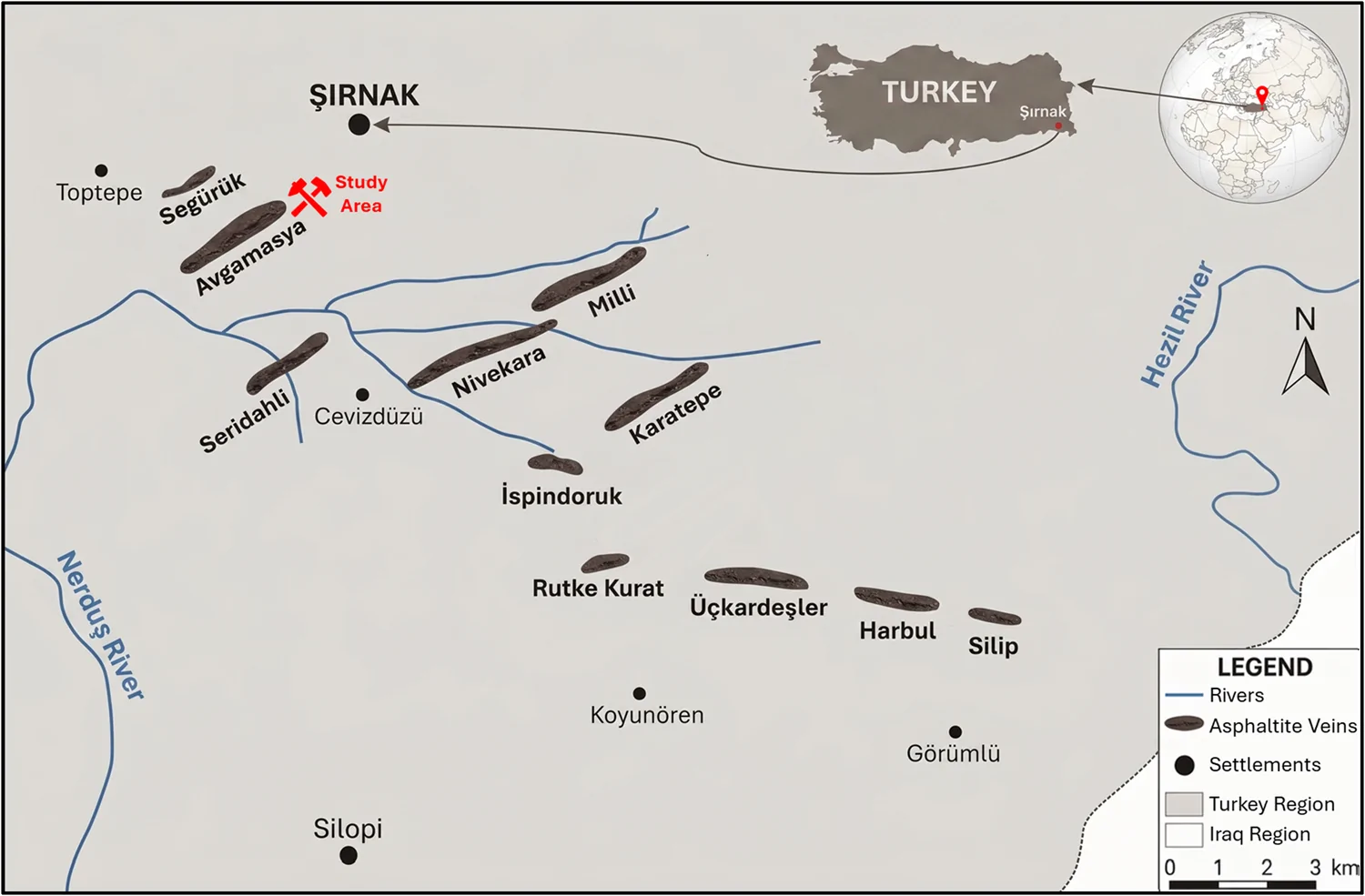
Geological location map of asphaltite deposits in the Şırnak–Avgamasya region, Turkey.

To enable its evaluation as a mineral filler in bitumen, the asphaltite sample was converted into ash and subjected to the following preparation procedures:

- The raw asphaltite was first crushed in an iron mortar until a fine powder was obtained.
- The powdered asphaltite was then incinerated in a muffle furnace at 1000 °C to produce AA through complete combustion of the organic components.
- The resulting ash was oven-dried at 105 °C for 24 h to remove any residual moisture.
- The dried ash was sieved through a No. 200 sieve (75 μm) to obtain a uniformly fine fraction suitable for incorporation into the binder.
- Finally, the processed AA was stored in sealed containers prior to mixing to prevent moisture absorption and possible carbonation.

The preparation procedure of AA is illustrated in Figure 2.

**Figure 2:**
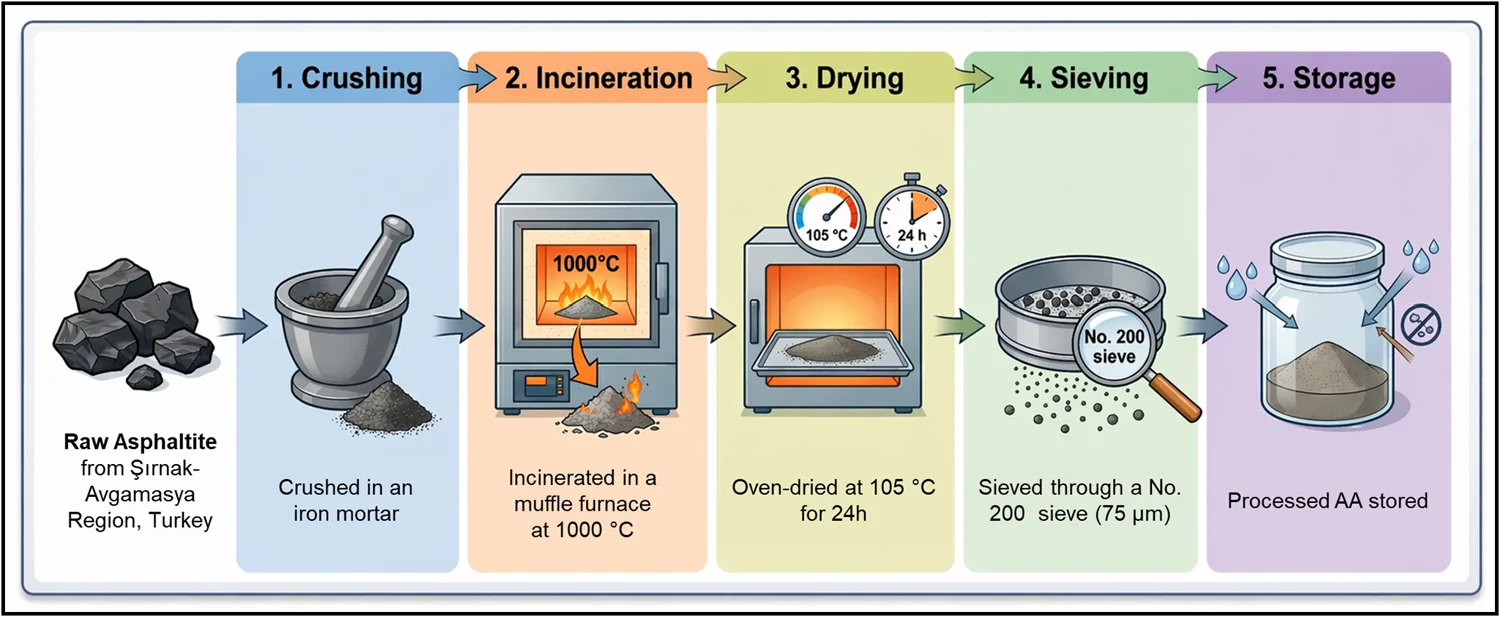
Workflow of AA preparation.

The morphological characteristics, elemental composition, and crystalline structure of the AA were examined through SEM-EDX, X-ray diffraction (XRD), FTIR, DSC and TGA respectively. A representative SEM micrograph of the AA particles is presented in Figure 3(a). The image shows that the particles possess an irregular and angular morphology with a heterogeneous surface structure. In addition, the particle surfaces appear relatively rough and include small microvoids and irregularities. Such morphological features are typical for ash-based materials produced through thermal processes and indicate a fragmented particle structure. Furthermore, the presence of smaller particles adhering to larger fragments suggests a certain degree of agglomeration. These rough and irregular surface characteristics are expected to increase the effective contact area, thereby enhancing the interaction between AA particles and the bitumen matrix.

**Figure 3:**
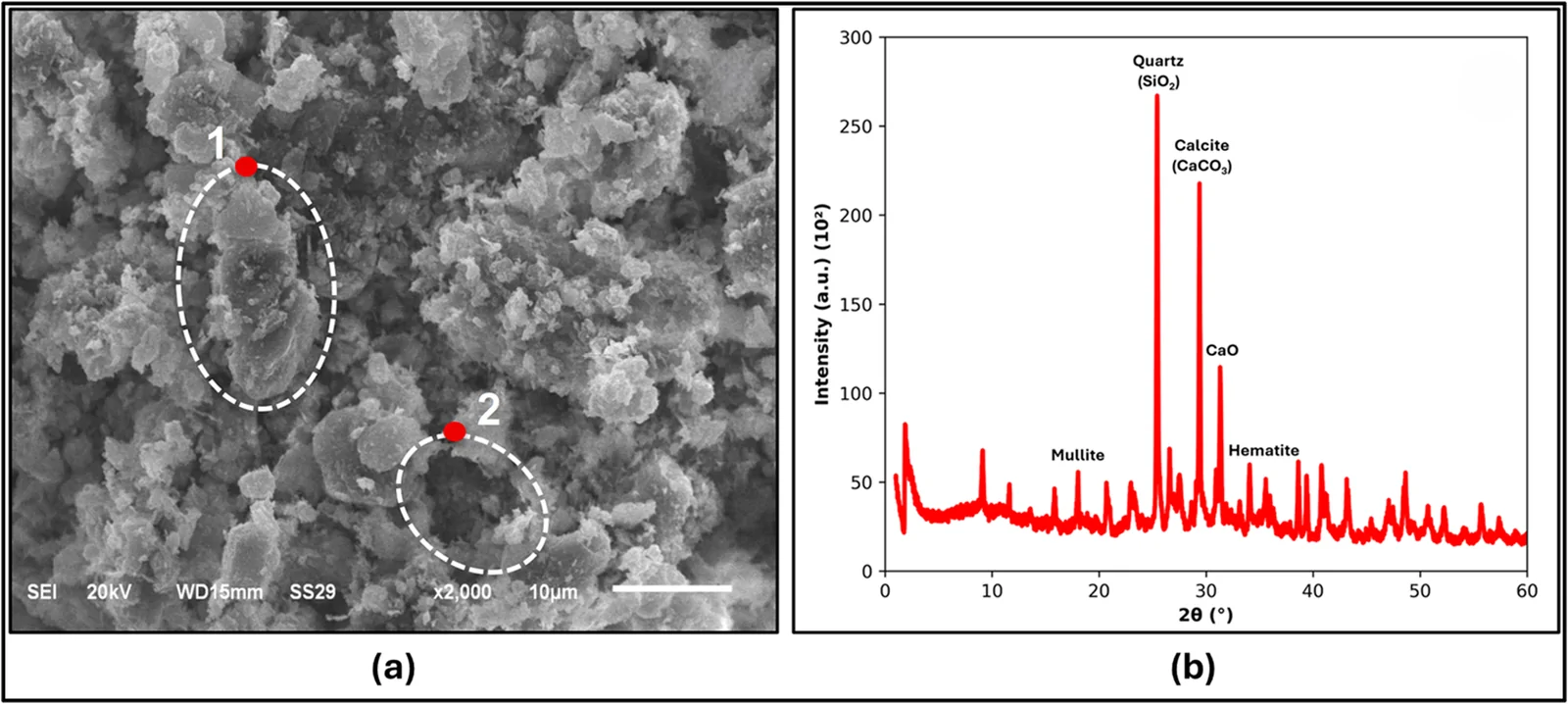
(a) SEM image [1. Agglomerated structures, 2. Micropores.] and (b) XRD analysis.

The EDX results shown in Table 2 indicate that the AA is mainly composed of oxygen and calcium, with contents of 28.87 wt % and 28.35 wt %, respectively. Significant amounts of sulfur (13.58 wt %), carbon (8.71 wt %), and silicon (9.23 wt %) were also detected, while aluminum, iron, potassium, and magnesium were present in moderate quantities. Minor trace elements with concentrations lower than 1 wt % were grouped under the category “Others”, accounting for approximately 3.38 wt % of the total composition. The high Ca and O contents suggest that the ash predominantly consists of calcium-based mineral compounds, whereas the presence of Si and Al indicates silicate and aluminosilicate phases. This elemental distribution is consistent with the mineral phases observed in the XRD analysis and supports the heterogeneous particle morphology revealed by the SEM images.

**Table 2:** Elemental Composition of AA Determined by EDX Analysis

Element	O	Ca	S	Si	C	Al	Fe	K	Mg	Pb	Others	Total
wt (%)	28.87	28.35	13.58	9.23	8.71	2.33	1.89	1.42	1.17	1.07	3.38	100

The crystalline structure of the AA was investigated using XRD, and the obtained diffractogram is presented in Figure 3(b). The pattern displays several diffraction peaks superimposed on a relatively broad background, indicating the coexistence of crystalline phases together with a partially amorphous component. A reflection observed at approximately 2θ ≈ 16–18° can be attributed to mullite, a common aluminosilicate phase frequently reported in combustion-derived ash materials. The most prominent peak appearing around 2θ ≈ 26° corresponds to quartz (SiO_2_). Additional peaks detected near 2θ ≈ 29–30° are associated with calcium-containing phases such as calcite (CaCO_3_), while weaker reflections observed between 30° and 35° may indicate the presence of other calcium-bearing or aluminosilicate minerals. Furthermore, a diffraction peak located near 2θ ≈ 35–36° can be related to hematite, suggesting the occurrence of iron-bearing mineral phases in the ash. The relatively broad background hump visible in the pattern indicates that a portion of the material exists in a disordered or glassy form, which is typical for ash generated during high-temperature thermal processes. In line with EDX analysis, these results collectively suggest that the AA is composed of quartz, calcium-based minerals, and minor aluminosilicate and iron oxide phases embedded within a partially amorphous matrix.

EDX analysis indicated a local Pb content of approximately 1.07 wt % in the analyzed AA sample. Although this finding alone is insufficient to assess its environmental significance or leaching behavior, further evaluation through quantitative bulk elemental analysis and standardized leaching tests may be considered for raw AA and AA-containing asphalt materials.

The FTIR spectrum of the AA, presented in Figure 4 indicates the presence of both residual organic functionalities and dominant inorganic mineral phases. A broad absorption band in the 3050–3400 cm^–1^ is associated with the stretching vibrations of hydroxyl groups (O–H), which can be linked to hydroxylated mineral surfaces.^25^ A weak band observed in the range of 2920 to 3000 cm^–1^ corresponds to the symmetric and asymmetric stretching of aliphatic C–H bonds. Its low intensity suggests that most organic constituents were removed during combustion, leaving only trace amounts of residual hydrocarbons.^26^ This observation is consistent with the EDX results, where inorganic elements such as Si, Ca, and Fe dominate while the carbon content remains relatively low.

**Figure 4:**
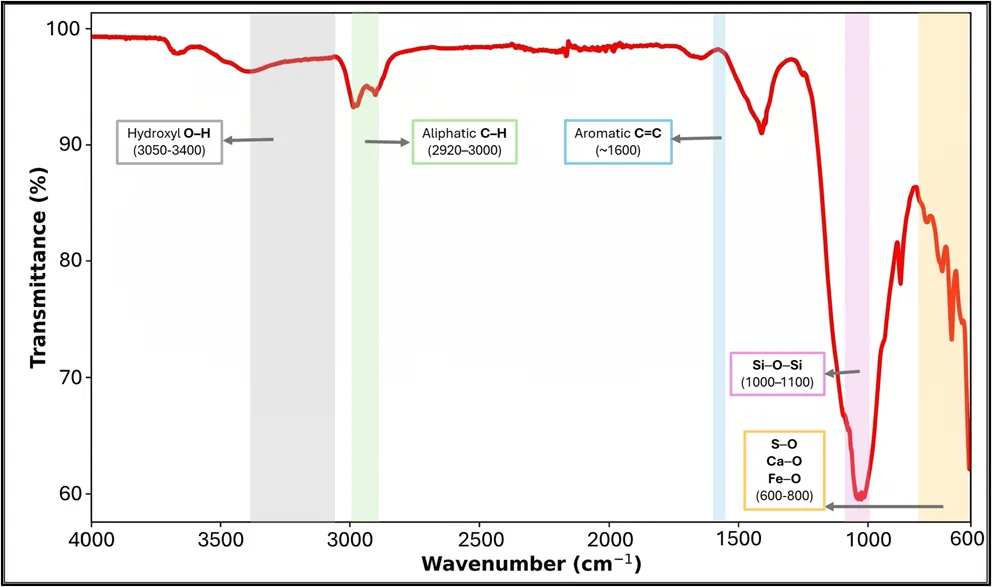
FTIR spectrum of AA showing the main functional groups and mineral-related vibrations.

A noticeable peak near 1600 cm^–1^ can be attributed either to C=C stretching in aromatic structures or to the bending mode of molecular water. In the case of AA, it is more likely related to residual aromatic carbon structures inherited from the parent material. The moderate intensity of this band further supports the predominantly inorganic character of the ash. The most intense absorption is observed between 1000–1100 cm^–1^ and is assigned to Si–O–Si asymmetric stretching vibrations.^27^ This feature clearly indicates the presence of silicate minerals, particularly quartz, which was also identified as the dominant phase in the XRD analysis. The strength of this band confirms the silica-rich composition of the ash. Additional bands in the lower wavenumber region, between 600 and 800 cm^–1^, are associated with metal–oxygen bonds such as Ca–O and Fe–O. These features support the presence of calcium and iron-bearing phases, including CaO and hematite, as identified in the XRD pattern and supported by the elemental composition obtained from EDX analysis. Considering the relatively high sulfur content obtained from EDX analysis, the weak absorptions observed in the ∼1100–1000 cm^–1^ and ∼600–700 cm^–1^ regions may also be partially associated with S–O stretching vibrations of sulfate species. However, the absence of distinct and sharp sulfate peaks suggests that sulfur is likely present in dispersed or amorphous forms or incorporated within complex mineral matrices rather than as well-crystallized sulfate phases. Overall, the FTIR results indicate that AA is primarily composed of inorganic mineral phases. The limited presence of aliphatic and aromatic groups points to the effective thermal decomposition of organic matter. These findings are in good agreement with SEM observations showing irregular and agglomerated particles, as well as with XRD and EDX analyses, confirming the mineralogical composition and chemical stability of the AA.

The thermal behavior of AA was evaluated using DSC and TGA, as presented in Figure 5(a,b), respectively. These analyses provide insight into both the thermal transitions and degradation characteristics of the material. The DSC thermogram in Figure 5(a) exhibits a broad glass transition region ranging from −41.32 °C to −12.45 °C, with a midpoint *T*_g_ of −24.87 °C. The relatively wide transition interval and low enthalpy change (Δ*H* = −1.94 J/g) indicate the absence of a well-defined polymeric phase and suggest a heterogeneous structure dominated by inorganic constituents. This weak thermal response reflects the limited presence of residual organic components within the ash. The TGA and derivative thermogravimetry (DTG) curves shown in Figure 5(b) further confirm the mineral-dominated nature of AA. The onset thermal degradation temperature (OTDT) was determined as 358.70 °C, while the maximum degradation rate occurs at 472.15 °C. These values indicate that only a limited fraction of thermally degradable material is present. The most notable result is the high residual mass of approximately 82.36%, which clearly demonstrates that the material consists predominantly of thermally stable inorganic phases. This high residue is also physically meaningful, as AA is obtained after combustion, meaning that most volatile and organic fractions have already been removed prior to testing, leaving behind a mineral-rich structure.

**Figure 5:**
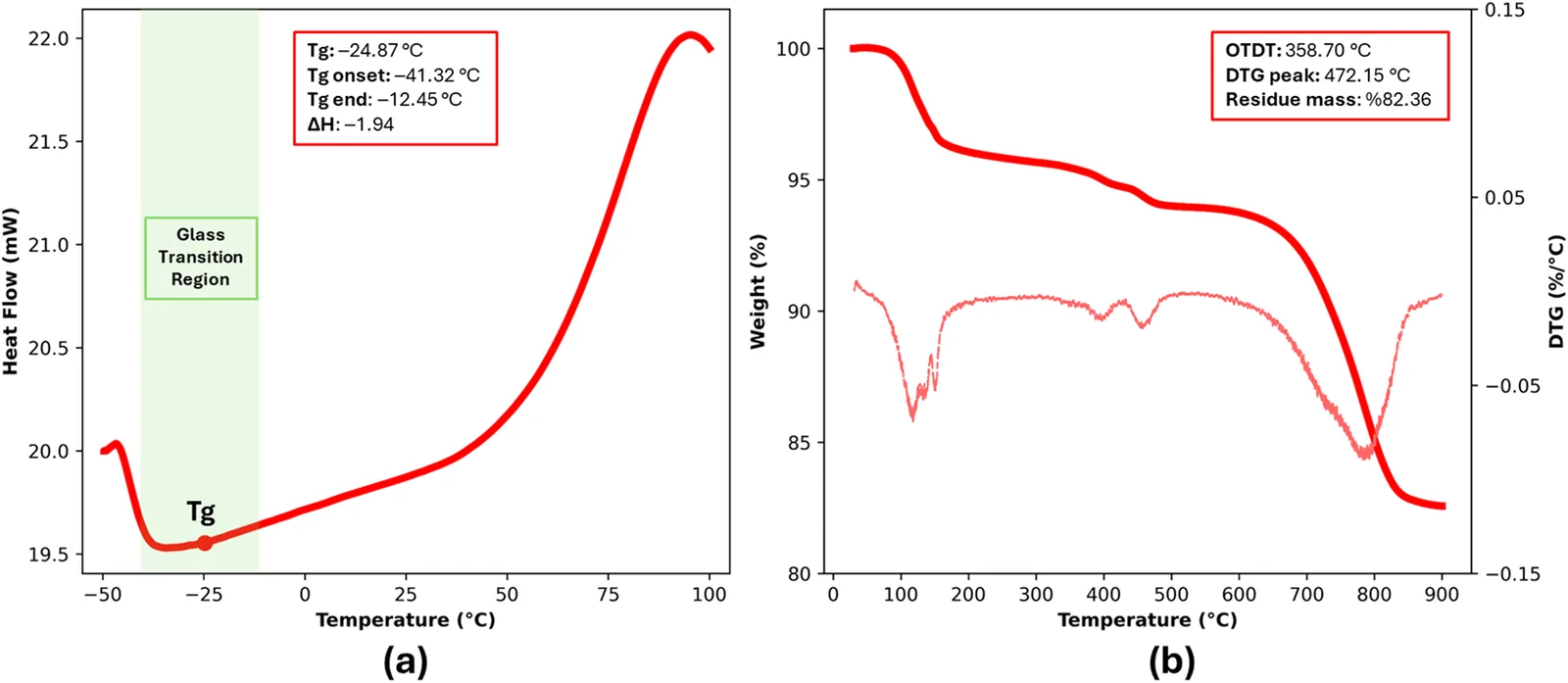
Thermal characterization of AA: (a) DSC thermogram showing the glass transition region (*T*_g_, onset, and end temperatures), and (b) TGA and DTG curves illustrating mass loss and degradation behavior.

Supporting characterization techniques are consistent with these findings. SEM observations revealed irregular and rough particles, XRD confirmed the presence of crystalline mineral phases such as quartz and Ca-based compounds, FTIR indicated dominant inorganic functional groups, and EDX analysis showed a composition rich in Ca, Si, and O. Overall, the DSC and TGA results indicate that AA exhibits limited thermal transitions but high thermal stability, primarily due to its inorganic and precombusted nature.

### 2.2 Experimental Program and Framework

This study establishes a systematic experimental framework to evaluate the influence of AA as a mineral filler in bituminous binders. The methodology integrates material characterization, preparation of AA-containing binders, and multilevel performance assessment to provide a comprehensive understanding of the physical interactions between AA particles and bitumen. A conventional B 50/70 penetration grade bitumen was selected as the base material, while AA was incorporated as the mineral filler. The overall study methodology is illustrated in Figure 6.

**Figure 6:**
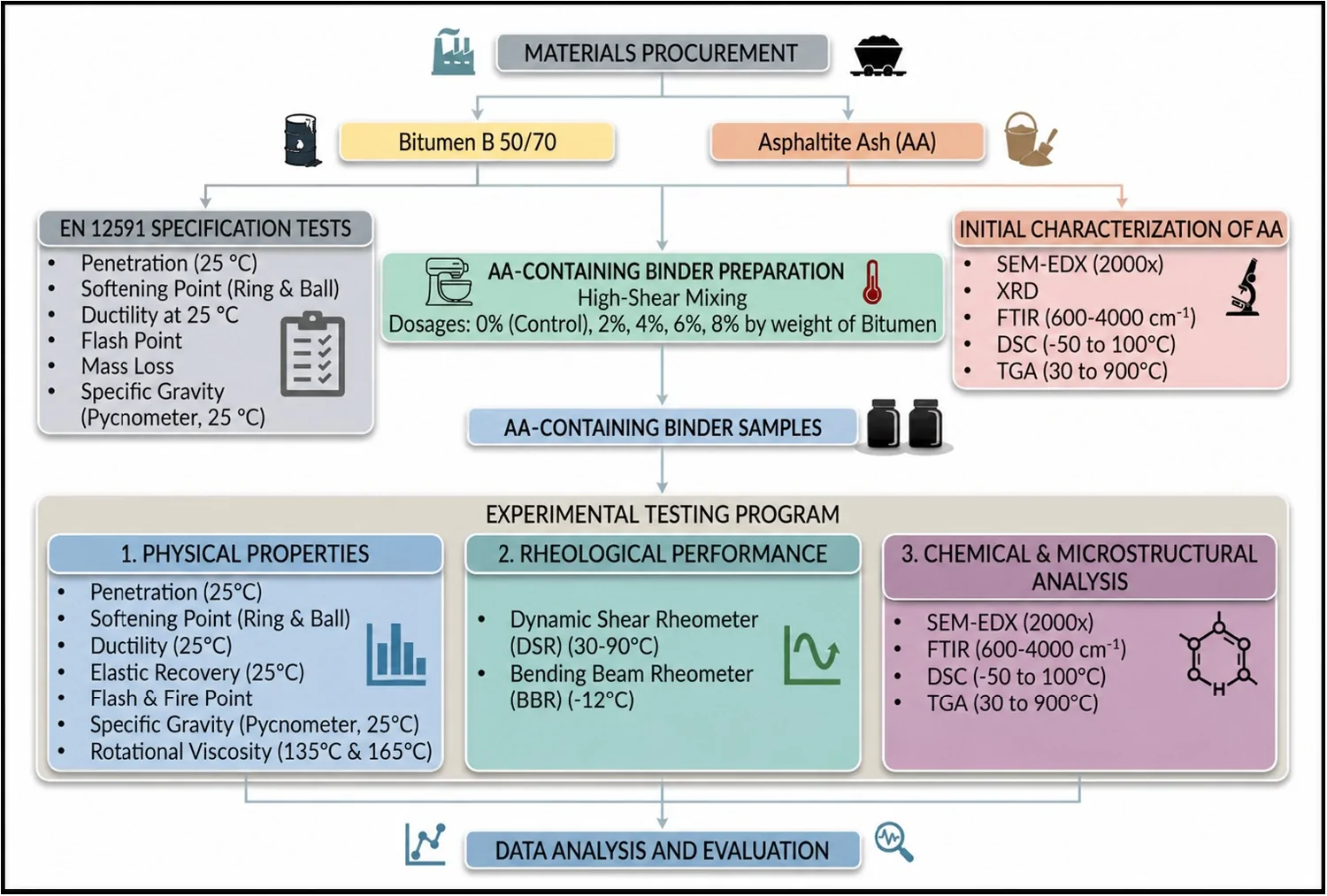
Flowchart of the experimental program.

### 2.3 Preparation of AA-Containing Binders

The neat binder was first conditioned at 165 °C for approximately 30 min to ensure full fluidity. AA was added in addition to the base-binder mass; therefore, the reported percentages were not normalized to a fixed total bitumen–AA blend mass of 100%. Accordingly, 20, 40, 60, and 80 g of AA were added to 1000 g of base binder to prepare AA2, AA4, AA6, and AA8, respectively, giving total batch masses of 1020, 1040, 1060, and 1080 g. These proportions were selected to examine potential improvements in performance while avoiding undesirable effects such as excessive viscosity increase, particle agglomeration, or reduced workability. The AA incorporation process is presented in Figure 7 with all its stages.

**Figure 7:**
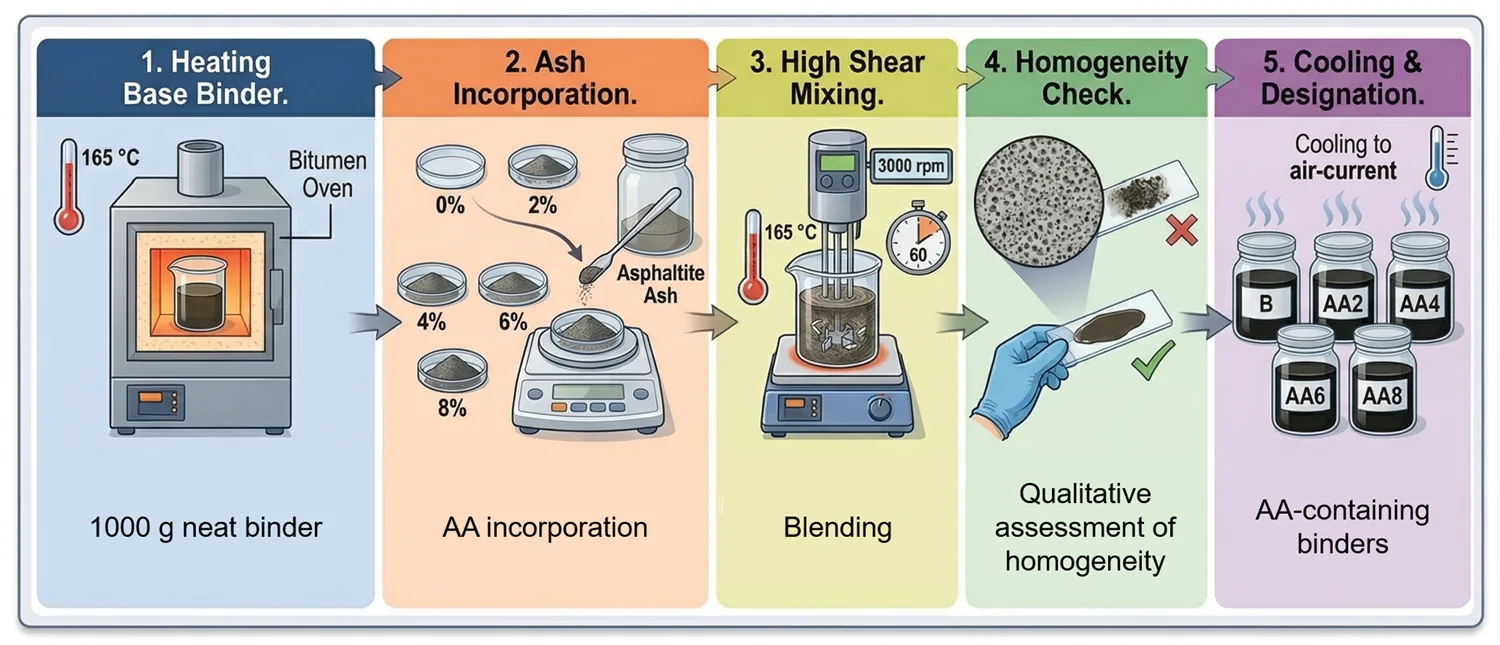
Schematic illustration of the incorporation of AA mineral filler into the base binder.

Blending was carried out using a high shear mixer operating at 3000 rpm for 60 min at 165 °C. This temperature was chosen to provide sufficiently low viscosity for effective particle dispersion without causing significant oxidative aging. The high-shear mixing procedure was used solely to obtain reproducible and initially well-dispersed laboratory-scale binder specimens for characterization and should not be interpreted as a required field-production method for incorporating AA. The application of high shear mixing facilitated a uniform distribution of ash particles within the binder matrix and helped limit particle clustering. For comparison purposes, the control binder was subjected to the same thermal and mechanical conditioning without the addition of AA, allowing the specific influence of the mineral filler to be isolated. Immediately after mixing, homogeneity was qualitatively assessed using a smear test.^28^ The AA-containing binders were then cooled to ambient temperature and stored in airtight containers until testing. The binders were designated as follows: B for the base binder, and AA2, AA4, AA6, and AA8 for binders containing 2, 4, 6, and 8% AA, respectively.

### 2.4 Physical Properties

Conventional properties of the binder were determined using standard test methods to ensure reliable and comparable results. Penetration at 25 °C (ASTM D5) was obtained by allowing a standard needle to penetrate the sample under a 100 g load for 5 s, with the measured depth (in 0.1 mm) indicating binder consistency.^29^ The softening point was measured according to ASTM D36 using the Ring-and-Ball method, where the temperature corresponding to a defined level of binder softening, sufficient for a steel ball to descend a specified distance, was recorded.^30^ Ductility at 25 °C was determined in line with ASTM D113 by stretching a briquette specimen at a constant rate until failure, and the elongation at break was taken as a measure of tensile deformability.^31^ Elastic recovery was evaluated following ASTM D6084 by measuring the extent to which a stretched specimen recovers its length after being cut, reflecting the elastic response of the binder.^32^ Specific gravity was determined using the pycnometer method described in ASTM D1480, providing the density of the binder relative to water.^33^ Viscosity measurements at 135 and 165 °C were carried out in accordance with ASTM D4402 using a rotational viscometer, offering an indication of flow behavior at typical mixing and compaction temperatures.^34^ Flash and fire points were determined based on ASTM D92 (Cleveland Open Cup), identifying the temperatures at which vapors briefly ignite and subsequently sustain combustion, respectively.^35^ All tests were conducted in duplicate, and average values were reported.

Temperature susceptibility of the binders was evaluated using the Penetration Index (PI) and Penetration Viscosity Number (PVN), which were calculated based on penetration, softening point, and viscosity measurements. The formulations of PI and PVN are presented in eqs ([Disp-formula eq1]) and ([Disp-formula eq2]), respectively. These indices provide a quantitative assessment of the binder’s sensitivity to temperature variations.

1 $$\mathrm{PI}=\frac{\left(1952-500\times \log\left({\mathrm{Pen}}_{\left(25^{{}^{\circ}}C\right)}\right)-20\times \mathrm{SP}\right)}{\left(50\times \log\left({\mathrm{Pen}}_{\left(25^{{}^{\circ}}C\right)}\right)-\mathrm{SP}-120\right)}$$

2 $$\mathrm{PVN}=-1.5\times \left(\frac{4.258-0.7967\times \log\left({\mathrm{Pen}}_{\left(25^{{}^{\circ}}C\right)}\right)-\log\left(V_{\left(135^{{}^{\circ}}C\right)}\right)}{0.795-0.1858\times \log\left({\mathrm{Pen}}_{\left(25^{{}^{\circ}}C\right)}\right)}\right)$$

where; Pen_(25°C)_ is the penetration value measured at 25 °C (0.1 mm), *V*_(135°C)_ is the rotational viscosity measured at 135 °C (Pa·s), and SP is the softening point temperature (°C).

### 2.5 Rheological Performance

#### 2.5.1 Dynamic Shear Rheometer (DSR)

The rheological properties of the binders at high and intermediate temperature were evaluated using a Dynamic Shear Rheometer (DSR) in accordance with ASTM D7175.^36^ The tests were conducted using a 25 mm parallel plate geometry with a 1 mm gap, under an angular frequency of 10 rad/s. Temperature sweep tests were performed to characterize the viscoelastic response of the binders over a range of temperatures. In this context, key rheological parameters including the complex shear modulus (G*), phase angle (δ), and rutting parameter (G*/sin δ) were determined. The failure temperature was identified as the temperature at which the rutting parameter satisfied the Superpave criterion of G*/sin δ ≥ 1.0 kPa for unaged binders, providing an indication of resistance to permanent deformation at elevated temperatures. For each variable, tests were conducted on two specimens, and the mean value was considered.

#### 2.5.2 Bending Beam Rheometer (BBR)

Low-temperature performance was assessed using the Bending Beam Rheometer (BBR). Before testing, the specimens were conditioned at −12 °C, a standard Superpave low-temperature reference commonly used to assess binder stiffness and stress relaxation characteristics.^37,38^ This test was used to evaluate the time-dependent creep behavior of the binders under constant load. The primary parameters obtained from the BBR test were the creep stiffness (S) at 60 s and the m-value, which represents the rate of change in stiffness with time. In addition, the S/m ratio was considered to provide further insight into the relationship between stiffness development and stress relaxation capacity. Together, these parameters were used to evaluate the influence of AA incorporation on the balance between increased stiffness and the binder’s ability to relieve thermal stresses at low temperatures. Each variable was evaluated using two samples, and the results were reported based on the average values obtained.

### 2.6 Chemical and Microstructural Analysis

To elucidate the chemical characteristics of the control and AA-containing binders, the following analyses were performed. These analyses enabled assessment of whether AA behaves predominantly as an inert mineral filler or exhibits potential chemical interactions within the bitumen matrix.

#### 2.6.1 SEM–EDX Analysis

SEM was employed to examine particle dispersion and microstructural evolution of AA-containing binders. Samples were gold-coated prior to imaging to prevent artifacts. EDX analysis was conducted to detect elemental composition attributable to AA and to confirm dispersion within the matrix.

#### 2.6.2 FTIR Spectroscopy

FTIR spectra were recorded in the 600–4000 cm^–1^ range to evaluate potential chemical interactions between AA and bitumen components. Peak shifts, intensity variations, and formation of new bands were analyzed to distinguish between physical blending and chemical mechanisms.

#### 2.6.3 Differential Scanning Calorimetry (DSC)

DSC analysis was performed under nitrogen atmosphere between −50 and 100 °C at a heating rate of 20 °C/min to determine glass transition temperature (Tg) and enthalpy changes.

#### 2.6.4 Thermogravimetric Analysis (TGA)

TGA was conducted from 30 to 900 °C under nitrogen atmosphere to evaluate thermal degradation behavior, onset degradation temperature, peak decomposition temperature, and residual mass.

## 3 Results and Discussion

### 3.1 Physical Properties

Conventional properties of both the control and AA-containing binders are presented in Figure 8. As shown in Figure 8(a), the penetration value gradually decreased with the incorporation of AA into the bitumen. This decline can primarily be attributed to the filler reinforcement effect, reduced molecular mobility, and the absorption of lighter bitumen components by AA. In line with the penetration results, the softening point temperatures of the AA-containing binders were higher than that of the neat B50/70, indicating an improved resistance to elevated temperatures before softening. Collectively, the observed increase in stiffness, as evidenced by the penetration and softening point results, suggests that AA-containing binders are more resistant to permanent deformation at high temperatures, however, this phenomenon should be further confirmed through rheological tests. The rotational viscosity results presented in Figure 8(b) indicate that AA-containing binders exhibited lower measured viscosity than the control binder at 135 °C, whereas AA incorporation increased viscosity at 165 °C. Given that AA was produced by combustion at 1000 °C and predominantly consists of inorganic phases, the decrease observed at 135 °C cannot be reliably attributed to residual hydrocarbons or maltene-like fractions. The present results do not establish the mechanism responsible for this temperature-dependent response; therefore, it is reported as an experimental observation without assigning a specific mechanism. Further measurements at different shear rates, together with storage-stability and particle-dispersion analyses, are required to clarify this behavior.

**Figure 8:**
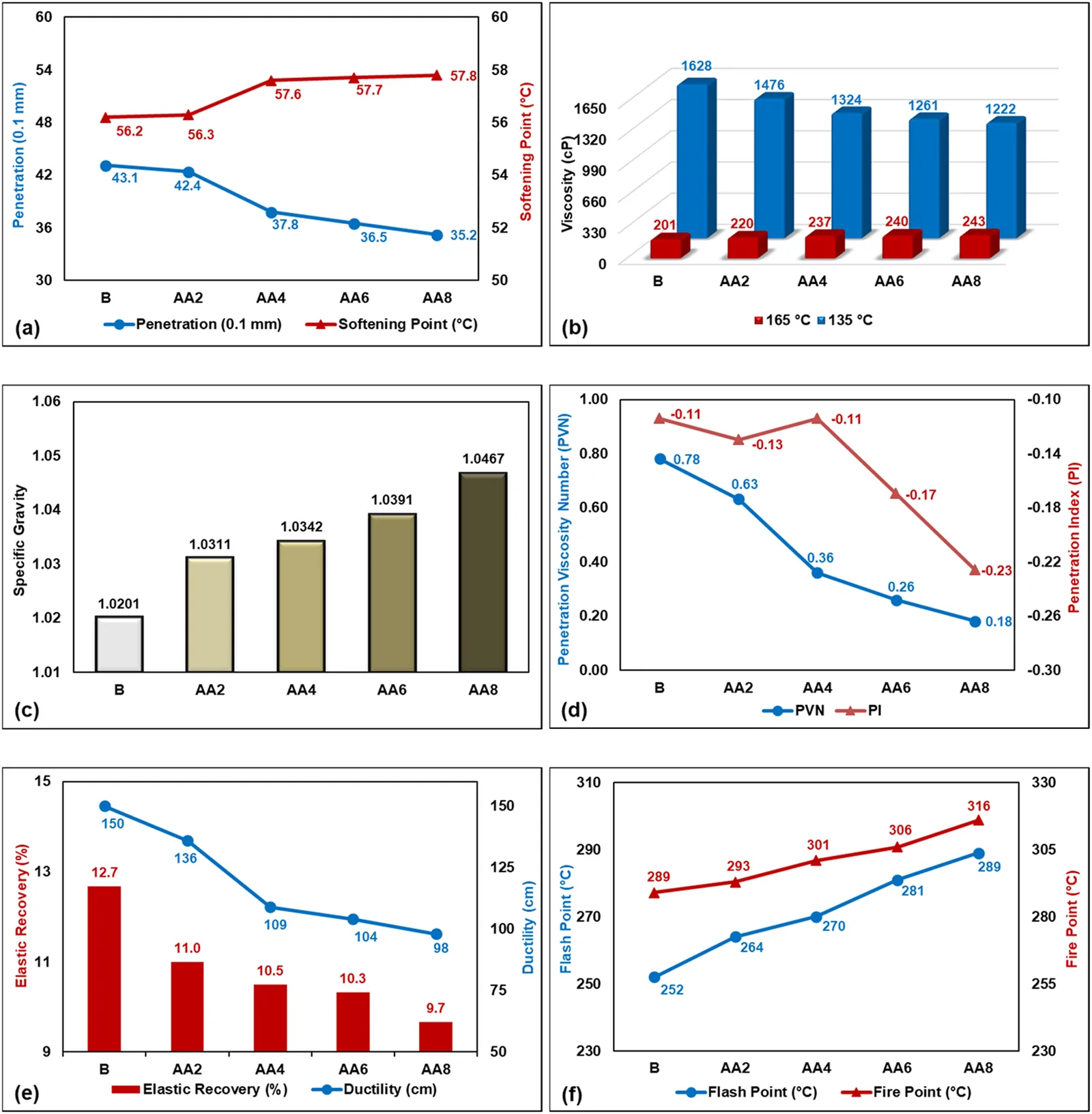
Physical properties of control and AA-containing binders: (a) penetration and softening point; (b) viscosity; (c) specific gravity; (d) PVN and PI; (e) elastic recovery and ductility; and (f) flash and fire points.

As previously demonstrated by SEM-EDX analysis, AA is mainly composed of dense and inorganic mineral particles. The presence of these heavier components increased the overall mass per unit volume of the neat binder, thereby raising the specific gravity as shown in Figure 8(c). Given that bitumen with higher specific gravity typically forms a denser and more stable structure, asphalt mixtures containing AA-containing binders are expected to better withstand permanent deformation at high temperatures. As clearly shown in Figure 8(d), the PVNs of the AA-containing binders were lower than that of the pure B50/70, which can be ascribed to the adsorption of the lighter fractions of bitumen by the porous AA particles. The decreased PVN reflects a stiffer, less deformable and more temperature-resistant bitumen, referring that rheological flow tendency of the AA-containing binders would be markedly reduced. A B50/70 bitumen typically possesses a PI in the range of −1 to 0, which is consistent with the value obtained in this study (−0.11) for both the control and the AA4 binder. As the AA content increased to 6% and 8%, the PI shifted slightly toward more negative values (−0.17 and −0.23, respectively). Nevertheless, all binders remained within the conventional PI range (−2 to +2), indicating that their temperature susceptibility remains acceptable for asphalt paving applications. The ductility values in Figure 8(e) elucidate that increasing AA dosage consistently curtailed the stretching capacity of the neat bitumen. As also proved by the decline in penetration and the rise in softening point, irregularly shaped AA grains with rough surface impeded the mobility of bitumen molecules and constituted a rigid internal framework within bituminous matrix. Parallel to this, elastic recovery of AA-containing binders regularly diminished. It is fairly recognized that bitumen’s elasticity is principally associated with asphaltene micelles and their interactions. It is previously stated that fine AA particles interrupted the continuity of the asphaltene network making bitumen harder and more resistant to flow. Compared to neat bitumen, elastic “spring like” part of AA-containing binders were accordingly weaker and they stored and released less elastic energy. As displayed in Figure 8(f), the presence of AA, already inert and nonvolatile, raised the flash point of the base bitumen. This effect is likely due to the confinement of light and volatile bitumen molecules within the dense ash formation, which diluted the free flammable components and consequently reduced the binder’s tendency to ignite at lower temperatures. The variation in fire points was in close agreement with that of the flash points.

### 3.2 Rheological Performance

#### 3.2.1 DSR Analysis

DSR test was conducted to further understand the role of AA in viscoelastic behavior of neat bitumen at medium to high temperatures. This test provided two principal parameters (complex modulus, G* and phase angle, δ) to evaluate the sandwiched binders’ resistance to deformation and their energy dissipation capability under sinusoidal loading. Complex modulus, consisting of storage modulus (*G*′) and loss modulus (*G*″), measures a bitumen’s total stiffness (elastic and viscous), while the phase angle represents the time delay between applied shear stress and resulting strain. The isochronal plots of G* and δ versus temperature domain (30–90 °C) are drawn in Figure 9(a,b), respectively. As expected, the G* values of both control and AA-containing binders decreased, while δ increased with rising temperature, pointing to a shift toward more viscous behavior. With its G* curve located at top of the graph, AA4 maintained the most elastic behavior, showing the highest G* values among all binders. At the 4% content, the more uniform distribution of AA particles appears to have produced a more effectively particle-reinforced structure, thereby restricting deformation and increasing the measured G*. This interpretation reflects physical filler reinforcement and does not imply the formation of a chemically cross-linked network.^39^ Compared to G*, changes in δ are generally more sensitive to a binder’s chemical structure, and, consequently, to AA incorporation. As shown in Figure 9(b), the base binder and AA2 binder exhibited relatively higher δ values corresponding to a viscous behavior transition, whereas the overlapping curves of AA4 and AA6 reflected nearly identical δ. Incorporation with 8% AA resulted in the lowest overall phase angle.

**Figure 9:**
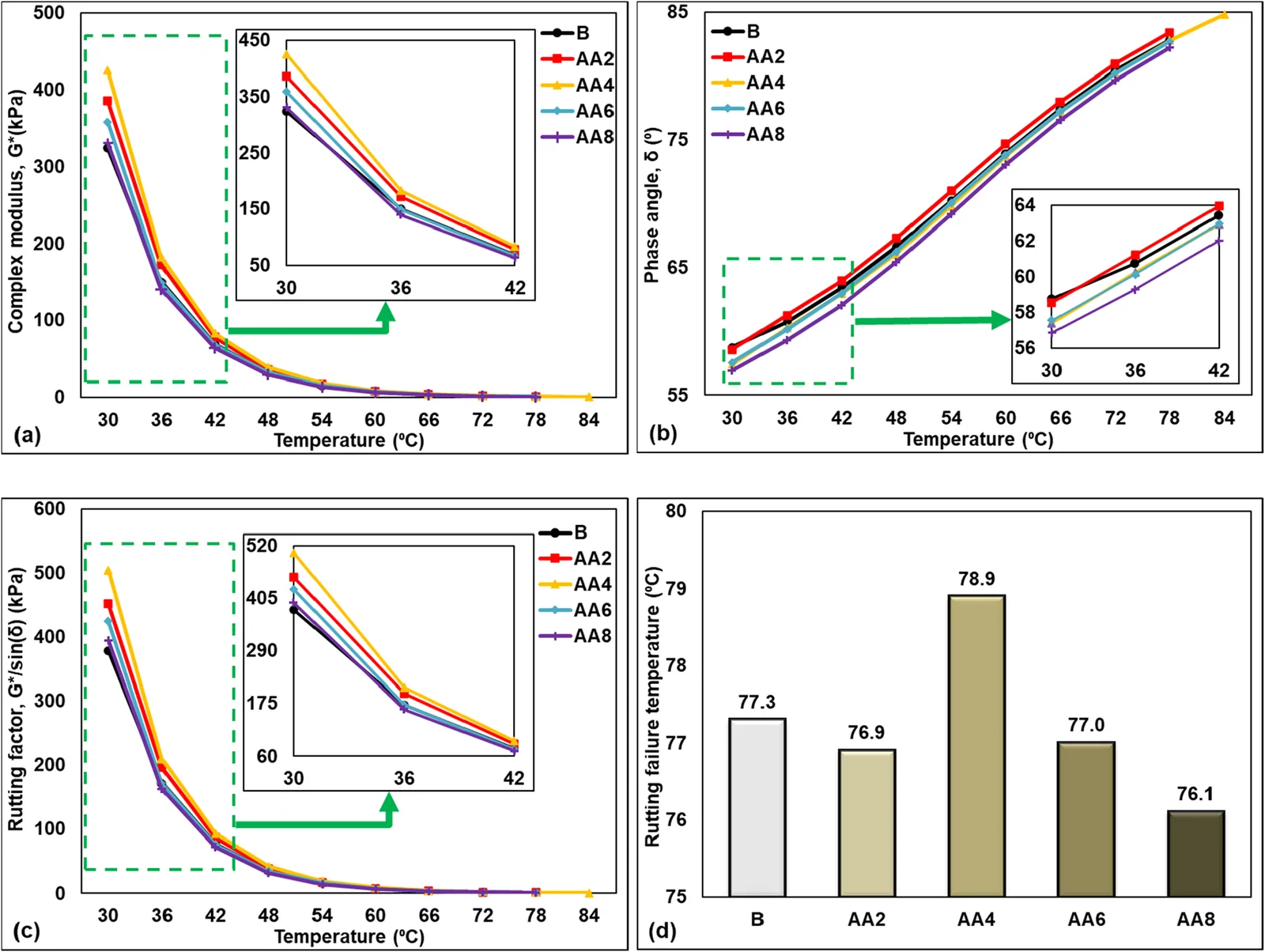
Viscoelastic analysis by DSR test: (a) complex modulus, (b) phase angle, (c) rutting factor, and (d) rutting failure temperature.

Rutting is a type of permanent pavement deformation, commonly appearing in the right lane and it is mainly caused by the loss of asphalt mixture’s stiffness under repeated heavy traffic loads and high-temperature conditions. Such distress creates irregularities on the asphalt pavement surface, reducing motorist’s steering control and impairing the stability of the vehicle at high speeds, on curved road sections, and during a sudden braking maneuver. Consequently, rutting, which significantly reduces the service life of the pavement, not only greatly diminishes driving comfort but also jeopardizes traffic safety by causing water accumulation and splashing during rainy conditions. To forestall binder-induced rutting, the rutting factor (G*/sin δ) of an unaged binder should be greater than 1.0 kPa at 1.59 Hz. The G*/sin δ curves of the control and AA-containing binders and their corresponding failure temperatures satisfying the rutting parameter specification are presented in Figure 9 (c), (d), respectively. The maximum rutting factor values at both medium and high temperatures were observed for the AA4 binder and its rutting failure temperature reached 78.9 °C. Given that this value was comparatively low for the control binder (77.3 °C), it may be concluded that incorporation of 4% AA meaningfully boosted the B50/70 bitumen’s resistance to plastic flow at high service temperatures. In line with the previously reported penetration and softening point results, this dosage exhibited a marked improvement in the rutting stiffness of the neat binder. The underlying cause could reside in the AA particles’ ability to absorb the lighter bitumen’s fractions, thereby transforming the bituminous matrix from a more sol-like (viscous and less structured) behavior to a more gel-like (elastic and more structured) state, and eventually restraining viscous flow at high temperatures. Compared with AA4, AA2, AA6, and AA8 exhibited lower G*/sin δ values, indicating lower high-temperature rutting resistance under the tested conditions, although only slight differences were observed in their rutting failure temperatures. Since G*/sin δ is a high-temperature rutting parameter for unaged binders, no conclusions regarding low-temperature or oxidative cracking resistance were drawn from these results.

#### 3.2.2 BBR Analysis

Thermal cracking remains one of the most critical challenges in flexible pavements under low-temperature conditions. The fundamental properties of bitumen, primarily its viscosity, glass transition temperature, and stiffness, play a deterministic role in an asphalt mixture’s resistance to low-temperature cracking. Bitumen mostly dissipates energy through permanent flow; however, as the temperature drops, bitumen’s this ability lessens and its stiffness increases, making asphalt pavement more brittle and susceptible to cracking. To assess and mitigate bitumen-driven low-temperature cracking, the BBR test is conducted to determine the binder’s stiffness (*S*) and creep rate (*m*-value). A decline in *S* coupled with an increase in *m*-value amounts to improved resistance of the bitumen to low-temperature cracking, thereby mitigating pavement distress in cold regions. The *S* and *m*-value of control and asphaltite binders at −12 °C are given in Figure 10(a,b), respectively.

**Figure 10:**
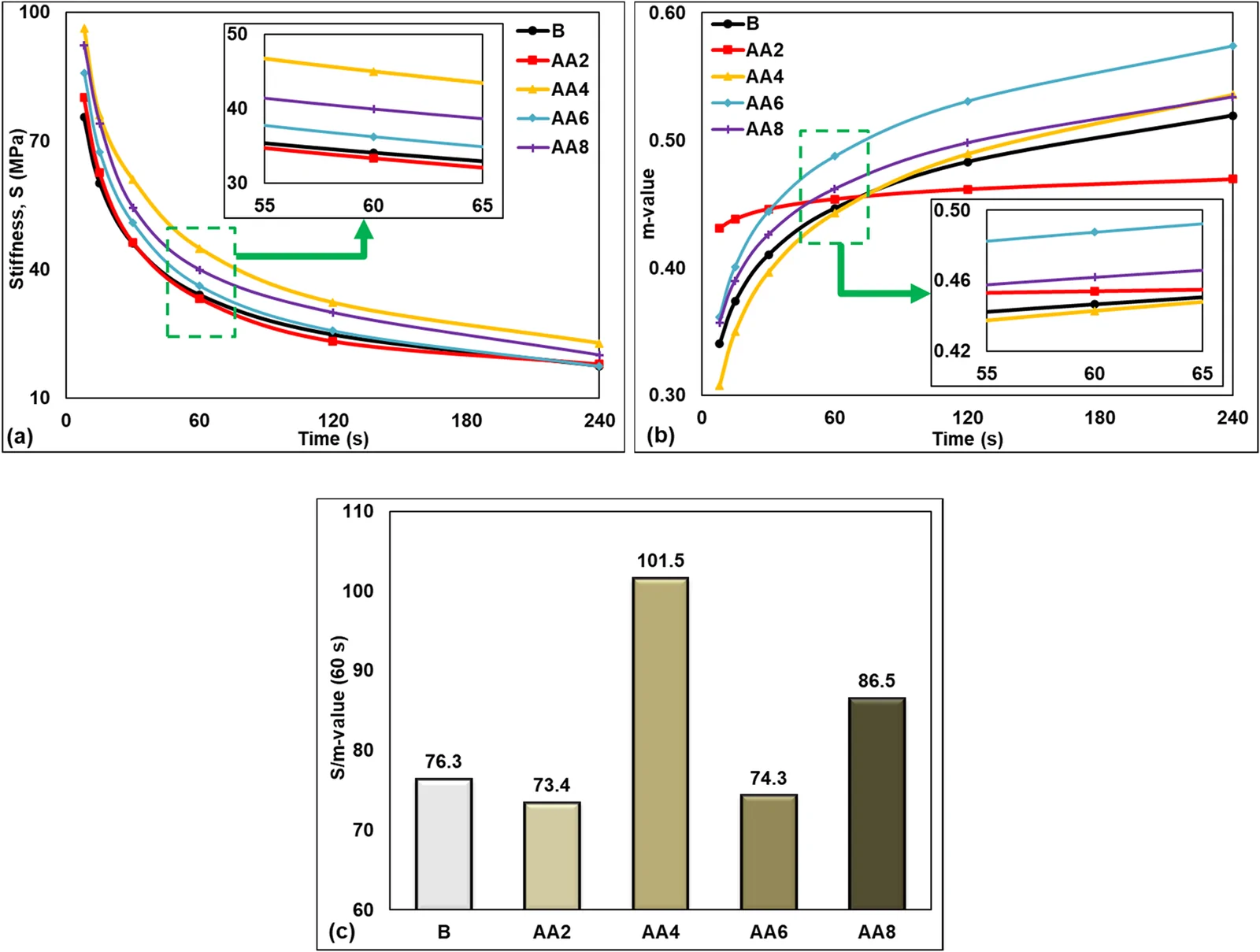
Viscoelastic behavior of samples at low temperature: (a) stiffness, (b) creep rate, and (c) the ratio of stiffness to creep rate.

As was expected, AA4 demonstrated the utmost stiffness with its highest *S* value. This finding pertinently accords with both previous conventional and DSR test results, and once again confirms that the incorporation of 4% AA increased the stiffness of the neat B50/70 bitumen. In contrast, a 2% dosage clearly enhanced low-temperature cracking resistance, as evidenced by the decline in creep stiffness. Consistent with the trend observed in the S value at 60 s, AA4 obviously exhibited the lowest *m*-value. However, the variation in *m*-values for the remaining samples was not parallel to that of the S values. Therefore, rather than relying solely upon either *S* or *m*-value, their combined evaluation offers a more fair and robust assessment of the viscoelastic response of bitumen in cold regions, as this approach rules out the effects of irregular variations in the low temperature creep behavior. A higher S/m ratio corresponds to a lower resistance to low-temperature cracking. The highest *S*/*m* value observed at the 4% AA dosage reflects a pronounced stiffening effect, which is associated with reduced resistance to low-temperature damage (Figure 10(c)). Conversely, the reduced S/m values, even those lower than that of the control bitumen, indicate that AA2 and AA6 would have a relatively greater capacity to dissipate energy, thereby protecting asphalt pavement from thermal contraction-driven cracking at subzero temperatures. To reiterate, rheological evaluation through DSR and BBR tests revealed that AA incorporation yields two distinct viscoelastic responses depending on the dosage. On the one hand, at the 4% level, it exclusively and substantially improved the virgin bitumen’s resistance to permanent deformation at medium and high service temperatures. On the other hand, the same control bitumen incorporated of either 2% or 6% AA maintained flexibility at low temperatures, allowing the asphalt pavement to expand and contract without cracking. Therefore, AA incorporation of 4% AA offers an effective solution for asphalt pavements in warm regions prone to permanent deformation, whereas 2% or 6% incorporation is promising to extend the service life of pavements in cold regions. However, it should be noted that these preliminary findings correspond to prior aging conditions, and further assessment is required to evaluate the modified binders’ ultimate performance after both short- and long-term aging.

### 3.3 Chemical and Microstructural Analysis

#### 3.3.1 SEM-EDX

The SEM images presented in Figure 11 illustrate the progressive evolution of the bitumen microstructure with increasing AA content. As seen in Figure 11(a), the neat binder (B) exhibits a relatively smooth and continuous surface, with no discernible solid inclusions or heterogeneities. This homogeneous appearance is typical for base bitumen and reflects the dominance of the viscous phase, where no rigid mineral phase interrupts the matrix continuity. With the incorporation of AA2, (Figure 11(b)), the initially smooth morphology begins to show the presence of discrete, finely dispersed particles. These particles appear sparsely distributed and partially embedded within the bitumen matrix, indicating a relatively good initial compatibility. At this dosage, the AA incorporation does not markedly disrupt the continuity of the binder phase but rather introduces localized heterogeneities that may contribute to slight stiffening effects without markedly altering the overall morphology. A more pronounced structural change is observed at AA4, as shown in Figure 11(c), where the number of particles increases and their interaction with the surrounding bitumen becomes more evident. The bitumen matrix appears locally distorted around the particles, forming wrinkled or folded regions. This behavior suggests a stronger physical interaction between the ash particles and the binder phase. Considering the previously identified rough, angular, and microporous morphology of AA particles, such deformation can be attributed to the mechanical interlocking and increased surface contact between the mineral particles and the bitumen. Rather than forming a chemical network, the system at this stage appears to develop a particle-reinforced structure, which can reasonably explain the enhanced stiffness and deformation resistance observed in rheological tests. As seen in Figure 11(d,e), the microstructure becomes increasingly heterogeneous at higher AA contents (AA6 and AA8). The particles are more numerous and tend to appear in clustered or agglomerated forms, consistent with the agglomeration tendency reported in the AA characterization. The distribution of particles becomes less uniform, and certain regions of the binder appear relatively particle-rich, while others remain comparatively smoother. This nonuniform dispersion suggests that beyond a certain dosage, the binder matrix may not be able to effectively accommodate and stabilize the added mineral phase.

**Figure 11:**
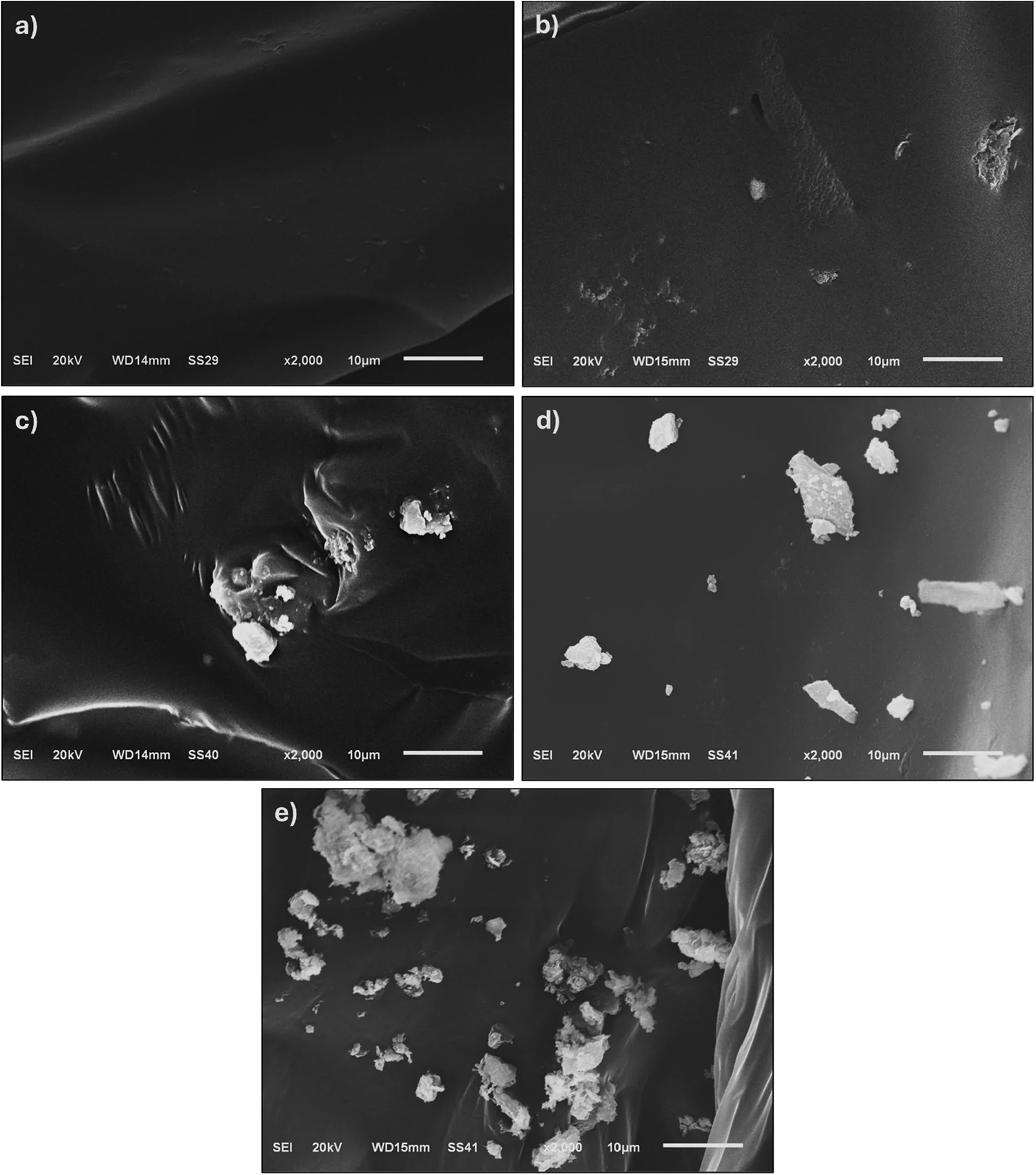
SEM images of neat and AA-containing binders: (a) B, (b) AA2, (c) AA4, (d) AA6, and (e) AA8.

The mineralogical composition of AA, dominated by calcium-based compounds, silica, and minor aluminosilicate and iron-bearing phases, helps to explain the observed morphological features. These predominantly inorganic and relatively rigid particles act as solid inclusions within the softer bitumen matrix. Their angular geometry and rough surfaces increase frictional resistance and limit the mobility of bitumen molecules in their vicinity. At moderate dosages (particularly 4%), this interaction appears beneficial, leading to a more structured and resistant microenvironment. However, at higher dosages, particle clustering and reduced dispersion efficiency may offset these advantages by introducing weak zones or stress concentration points within the binder. Overall, the SEM observations indicate that AA functions as a mineral filler through physical particle–binder interactions rather than chemical bonding. The transition from a homogeneous binder matrix to a particle-reinforced system was clearly dependent on AA content. Moderate contents produced a comparatively more uniform particle distribution, whereas higher contents increased heterogeneity and agglomeration, which may reduce the consistency of the binder response.

The EDX results (Table 3) show that the neat binder is largely dominated by carbon, reflecting its hydrocarbon-based structure, with only limited inorganic content. With the addition of AA, a gradual reduction in carbon is observed alongside a noticeable increase in mineral-related elements. In particular, Ca and Si display a consistent upward trend, which aligns with the previously identified mineral composition of the ash. Elements such as Al, Fe, K, and Na, which are not present in the neat binder, appear in the AA-containing samples and contribute to a more complex elemental structure. Sulfur remains relatively stable across all binders, suggesting contributions from both the base bitumen and the ash. Overall, the results indicate that AA enriches the binder with a variety of inorganic constituents, leading to a more mineral-rich and heterogeneous system.

**Table 3:** Elemental Composition of Control and AA-Containing Binders Obtained from EDX Analysis, Showing the Variation in Major and Trace Elements with Increasing AA Content

Element (wt %)	B	AA2	AA4	AA6	AA8
C	77.854	74.869	73.974	74.809	73.288
O	8.899	8.970	9.196	9.133	8.740
S	12.914	12.593	12.849	12.099	13.652
Mg	0.180	0.181	0.154	0.117	0.099
Ca	0.153	0.397	0.518	0.492	0.662
Si	-	0.895	1.173	1.151	1.473
Al	-	0.261	0.243	0.293	0.209
Fe	-	0.274	0.276	0.296	0.260
K	-	0.264	0.236	0.365	0.265
Pb	-	0.237	0.244	0.227	0.214
Na	-	0.131	0.161	0.255	0.210
V	-	0.178	0.178	0.144	0.167
Co	-	0.200	0.155	0.165	0.194
Ni	-	0.260	0.291	0.187	0.241
Cu	-	0.290	0.352	0.267	0.326
Total	100.000	100.000	100.000	100.000	100.000

#### 3.3.2 FTIR

As shown in Figure 12, the FTIR spectra of the neat and AA-containing binders reveal that the fundamental chemical structure of the bitumen remains largely unchanged after AA incorporation, as indicated by the consistent positions of the main absorption bands across all samples. The spectra are dominated by characteristic hydrocarbon-related vibrations, with prominent bands observed in the aliphatic C–H stretching region at approximately 2920–2850 cm^–1^, confirming the preservation of the binder’s hydrocarbon backbone. The band in the range of 1580–1620 cm^–1^ corresponds to aromatic C=C stretching, indicating that the aromatic framework is retained. In the intermediate region, bands near 1450–1375 cm^–1^ are attributed to bending vibrations of CH_2_ and CH_3_ groups, whereas minor features around 1300–1250 cm^–1^ are associated with deformation vibrations and limited oxygen-containing functionalities. These bands show minimal variation, suggesting that the organic phase of the binder does not undergo significant chemical alteration. More pronounced changes are observed in the 1000–1100 cm^–1^ region, where an increase in band intensity with increasing AA content is evident. This band is assigned to Si–O–Si asymmetric stretching vibrations, reflecting the incorporation of silicate minerals, in agreement with XRD and EDX findings. Additional contributions from S–O and metal–oxygen bonds (Ca–O, Fe–O) are also likely in the lower wavenumber region (800–600 cm^–1^), further confirming the presence of inorganic phases within the binder matrix.

**Figure 12:**
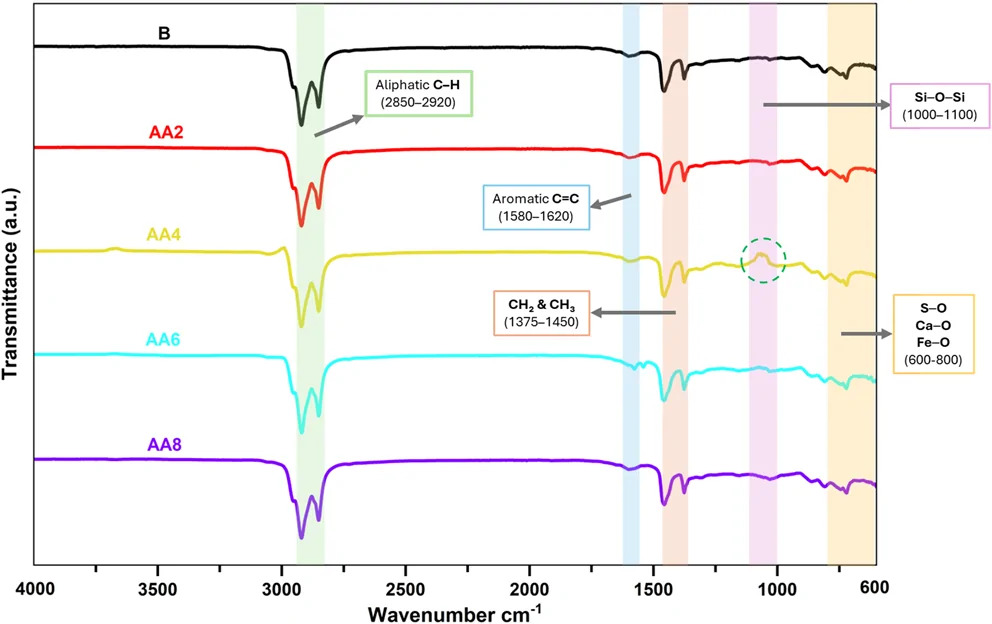
FTIR spectra of neat and AA-containing binders.

Among all samples, AA4 exhibits a more pronounced increase in the intensity of mineral-related bands, particularly in the 1000–1100 cm^–1^ region, suggesting a more effective interaction between the bitumen matrix and AA particles at this dosage. This observation is consistent with SEM results, where AA4 showed a more homogeneous dispersion and stronger particle–matrix interaction. Importantly, this behavior is also supported by rheological and physical test results. DSR analysis indicates that AA4 exhibits the highest complex modulus (G*) and rutting parameter (G*/sin δ), reflecting enhanced stiffness and resistance to permanent deformation at elevated temperatures. In terms of conventional properties, the AA4 sample exhibited a noticeable increase in softening point compared to the other samples, along with clear reductions in penetration and ductility values, indicating a stiffer binder with enhanced resistance to high temperatures.

Taken together, these results suggest that the enhanced mineral-related FTIR response observed for AA4 is not merely a spectral feature, but rather a manifestation of improved microstructural integration and optimized particle dispersion. This leads to a more effective reinforcement of the binder matrix, which is directly reflected in its superior rheological performance. In contrast, higher dosages such as AA6 and AA8 do not show a proportional increase in band intensity, likely due to particle agglomeration and reduced dispersion efficiency, as observed in SEM images, which limits their contribution to mechanical performance. The absence of new characteristic peaks across all spectra confirms that no new characteristic bands attributable to chemical reactions were observed during incorporation. Instead, the changes observed in FTIR are primarily associated with the physical incorporation of mineral phases, which is further supported by EDX results showing increased concentrations of Ca, Si, and Al without altering the dominant hydrocarbon composition. Overall, the FTIR analysis demonstrates that AA acts as a mineral filler acting through physical mechanisms. The combined evidence from FTIR, SEM, EDX, and rheological analyses indicates that the effectiveness of the physical filler action is strongly dependent on particle dispersion, with 4% AA providing the most balanced and effective enhancement of binder performance.

#### 3.3.3 DSC

The glass transition temperature (*T*_g_) values were determined from the DSC thermograms using the tangent method. In this approach, tangents are drawn to the baseline before the transition and to the steepest slope of the heat flow curve within the transition region. The intersection of these two tangents is defined as the *T*_g_ value. In addition, the onset and end temperatures of the glass transition were obtained by identifying the intersections of the extrapolated baseline with the tangent drawn at the inflection region. This procedure allows a more consistent and reproducible determination of the transition limits, particularly for complex materials such as bitumen. The enthalpy change (Δ*H*) associated with the glass transition was calculated by integrating the area under the DSC curve within the transition region. This calculation was performed by subtracting the baseline from the measured heat flow signal and integrating the resulting curve over the temperature range between *T*_g_ onset and *T*_g_ end.^40,41^ Mathematically, the enthalpy change can be expressed as given in eq 3 below

3 $$\Delta H=\int_{T_{onset}}^{T_{end}}\mathrm{q̇}\left(T\right)dT$$

where; *q̇*(*T*) represents the heat flow as a function of temperature.

The DSC thermograms and corresponding quantitative results indicate that the incorporation of AA leads to a systematic shift in the thermal transition behavior of the binder. As shown in Figure 13, all AA-containing binders exhibit a slight but consistent decrease in Tg compared to the neat binder. The Tg value decreases from −25.54 °C (B) to −29.20 °C (AA4), while AA6 and AA8 show similar but slightly less pronounced reductions. This shift toward lower temperatures suggests an increase in molecular mobility at low temperatures, associated with changes in the internal structure of the binder matrix. The glass transition region, extending approximately from −40 °C to −10 °C, shows a moderate broadening with the addition of AA, as reflected by the Tg onset and Tg end values presented in Table 4. In particular, AA4 exhibits the lowest onset temperature (−43.19 °C) together with a relatively higher end temperature (−10.07 °C), indicating a wider transition interval compared to the neat binder. From a thermodynamic perspective, the Δ*H* values remain relatively stable across all samples, ranging between −2.17 and −2.29 J/g. This limited variation suggests that the incorporation of AA is associated with only limited variation in the energy associated with the glass transition process. This observation supports the FTIR results, where no new functional groups were identified, and is consistent with EDX findings indicating increased mineral content without fundamental changes in the organic composition of the binder.

**Figure 13:**
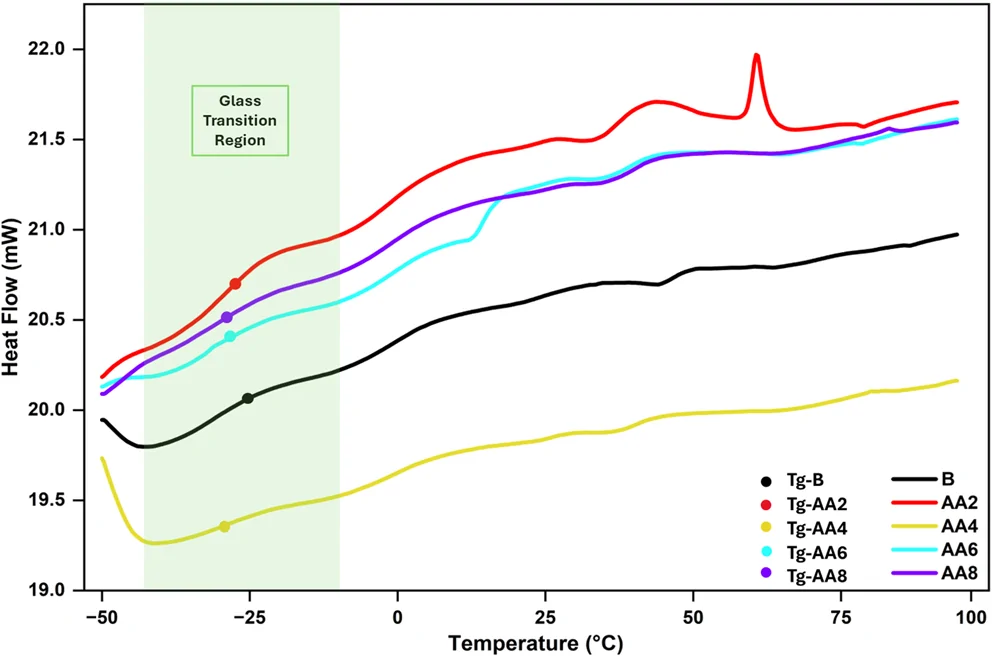
DSC thermograms of neat and AA-containing binders. The green shaded area represents the glass transition region, within which Tg values were determined using the tangent method. The thermograms illustrate the shift in glass transition behavior with increasing AA content.

**Table 4:** DSC-Derived Glass Transition Parameters of Neat and AA-Containing Binders

Sample	*T*_g_ (°C)	*T*_g_ onset (°C)	*T*_g_ end (°C)	Δ*H* (J/g)
B	–25.54	–39.96	–13.08	–2.29
AA2	–27.78	–41.27	–14.55	–2.17
AA4	–29.20	–43.19	–10.07	–2.17
AA6	–28.64	–42.81	–13.21	–2.20
AA8	–29.06	–42.70	–14.28	–2.25

Among all samples, AA4 exhibits the most pronounced thermal response, characterized by the lowest Tg and the widest glass transition range. These results indicate a more effective interaction between the binder and AA at this dosage level. As previously discussed, the trends observed in physical and rheological properties are in agreement with this behavior, further supporting the influence of this AA content. In contrast, higher AA contents (AA6 and AA8) do not lead to further improvements in thermal transition behavior, suggesting that increasing the dosage beyond this level does not enhance magnitude of the thermal response. Overall, when evaluated solely based on DSC results, the AA4 sample demonstrates the most favorable thermal performance, indicating that this dosage provides an optimal balance in terms of glass transition characteristics.

#### 3.3.4 TGA

Thermal degradation parameters were determined from the TGA and DTG curves using standard interpretation procedures. The OTDT was identified from the TGA curve as the temperature at which the initial deviation from the baseline occurs, corresponding to the beginning of significant mass loss. The DTG peak temperature was obtained from the minimum point of the DTG curve, representing the maximum rate of decomposition. The residue mass (%) was determined from the remaining mass at the end of the test. These values were extracted and quantified for all samples and are summarized in Table 5.

**Table 5:** Thermal Degradation Parameters Obtained From TGA Analysis

Sample	OTDT (°C)	DTG peak value (°C)	Residue mass (%)
B	345.27	468.34	10.23
AA2	352.18	472.11	12.79
AA4	360.42	478.56	16.87
AA6	355.63	474.29	18.68
AA8	350.91	471.37	21.31

As presented in Figure 14 and Table 5, all binders exhibit a single dominant degradation stage, primarily occurring within the temperature range of approximately 400–500 °C. The neat binder shows an OTDT of 345.27 °C, while the AA-containing binders display a gradual increase in this value up to AA4 (360.42 °C), indicating an improvement in thermal stability with the incorporation of AA. A similar trend is observed for the DTG peak temperatures, where AA4 reaches the highest value (478.56 °C), reflecting a delayed and more stable degradation process compared to the other samples.

**Figure 14:**
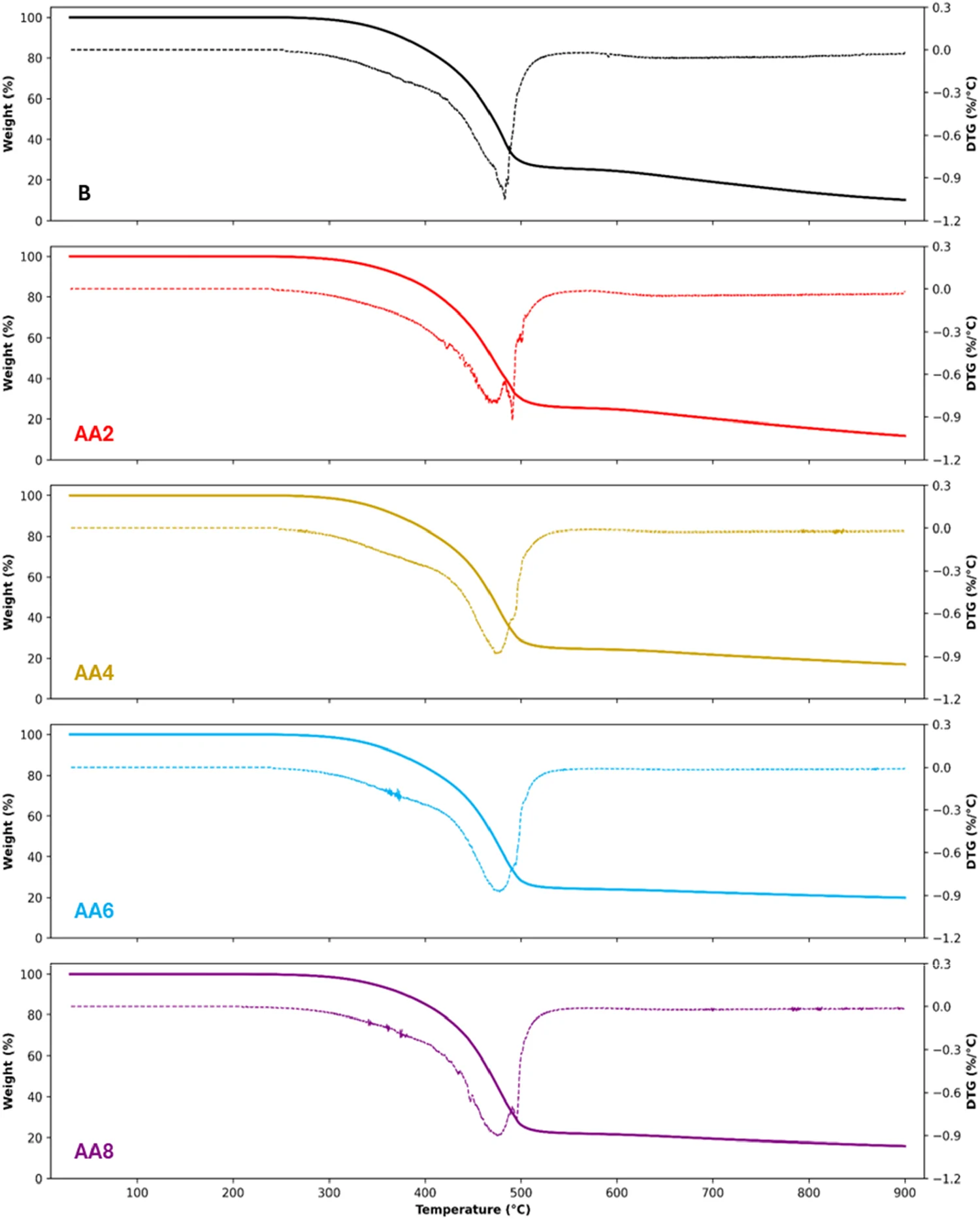
TG and DTG curves of neat and AA-containing binders. The solid lines represent the mass loss (weight, %) as a function of temperature, while the dashed lines correspond to the DTG curves, indicating the rate of mass change (%/°C).

Among all formulations, AA4 demonstrates the most favorable thermal behavior, exhibiting both the highest OTDT and DTG peak temperature. This suggests that the interaction between the bitumen matrix and AA is most effective at this dosage, leading to enhanced resistance against thermal decomposition. This observation is consistent with previous findings from SEM, FTIR, and rheological analyses, where AA4 was identified as the optimum incorporation level in terms of dispersion and performance balance. At higher additive contents (AA6 and AA8), a slight reduction in OTDT and DTG peak values is observed, indicating that increasing AA content beyond 4% does not further improve thermal stability. This behavior can be attributed to possible particle agglomeration and reduced dispersion efficiency, as previously discussed. Furthermore, the residue mass values show a clear increasing trend with increasing AA content, rising from 10.23% for the neat binder to 21.31% for AA8. This increase is directly associated with the presence of inorganic mineral constituents within the AA, which do not decompose within the tested temperature range. The gradual increase in residual mass confirms the contribution of these mineral phases to the overall thermal behavior of the AA-containing binders. Overall, the TGA results indicate that AA enhances the thermal stability of the binder up to an optimal level, with AA4 providing the most effective improvement, while higher dosages result in diminishing returns.

## 4 Conclusions and Recommendations

Mining wastes pose significant environmental challenges due to their accumulation and limited disposal options; however, their effective reutilization in engineering materials offers a waste-valorization pathway that reduces environmental impact while enhancing material performance. This study investigated the feasibility of utilizing AA as a mineral filler in bituminous binders through a comprehensive evaluation combining physical, rheological, and chemical analyses. The results consistently demonstrated that AA alters the binder structure primarily through physical interactions, leading to notable changes in performance. The findings highlight that an optimum balance between stiffness, thermal stability, and workability can be achieved at a specific additive content.

- The incorporation of AA resulted in a decrease in penetration (18.3%) and ductility (34.7%), accompanied by an increase in softening point (∼3%) and viscosity at 165 °C (20.9%), indicating a transition toward a stiffer and more temperature-resistant binder structure.
- The high-temperature rheological response varied with AA content. Among the tested formulations, AA4 exhibited the highest measured rutting failure temperature (78.9 °C), approximately 1.6 °C higher than that of the neat binder, representing the most favorable high-temperature response under the tested conditions. However, further increases in dosage led to a slight decline, likely due to reduced dispersion efficiency. In contrast, BBR results indicated that although stiffness increased with AA content, improvements in stress relaxation were limited, particularly AA4 and AA8, resulted in a more stiffness-dominated response, which may negatively affect low-temperature performance.
- FTIR results indicated that no new chemical functional groups were formed, confirming that the interaction mechanism is predominantly physical rather than chemical. Notably, AA4 exhibited a more pronounced intensity in the 1000–1100 cm^–1^ region, associated with Si–O–Si vibrations, suggesting a more effective incorporation of mineral phases into the binder matrix. This observation is consistent with SEM findings, where AA4 showed a more uniform particle distribution and improved particle–matrix interaction compared to higher dosages, which tended to exhibit signs of agglomeration.
- Thermal analyses (DSC and TGA) showed that AA enhances thermal stability, with higher degradation temperatures and increased residual mass, reflecting the contribution of mineral phases; AA4 again provided the most balanced thermal response.
- Overall, 4% AA addition (AA4) was identified as the optimum interaction level, offering the most favorable combination of physical properties, rheological performance, and thermal stability without compromising workability.
- The utilization of AA as a mineral filler may contribute to the valorization of this industrial byproduct; however, its environmental performance relative to conventional fillers requires verification through a comparative life-cycle assessment.

From a practical perspective, future studies should extend this work to the mixture level, focusing on asphalt mixtures incorporating AA-containing binders. Investigations on mechanical performance, moisture susceptibility, and long-term durability will be essential to fully assess the applicability of AA in pavement engineering.

## Data Availability

The data presented in this article are available on request from the corresponding author.
